# Behaviour and Dispersal of Mobile Salmon Lice When Detached From the Host

**DOI:** 10.1111/jfd.14143

**Published:** 2025-05-10

**Authors:** Luke T. Barrett, Mari F. Jensen, Sussie Dalvin, Frode Oppedal

**Affiliations:** ^1^ Sustainable Aquaculture Laboratory – Temperate and Tropical (SALTT), Queenscliff Marine Science Centre Deakin University Victoria Australia; ^2^ Oceanography and Climate Institute of Marine Research Bergen Norway; ^3^ Bjerknes Centre for Climate Research Bergen Norway; ^4^ Pathogens and Disease Transfer Institute of Marine Research Bergen Norway; ^5^ Animal Welfare Research Group Institute of Marine Research Matredal Norway

**Keywords:** dispersal, host finding, parasite behaviour, reinfection, reinfestation, salmon aquaculture

## Abstract

Sea lice can flourish when salmon are farmed in open sea‐cages, necessitating treatments to control outbreaks and reduce larval export. However, mobile ectoparasitic stages can be dislodged during crowding or other procedures, and potentially reinfest farmed or wild fish. We studied vertical movements and host‐finding behaviours of mobile salmon lice ( *Lepeophtheirus salmonis* ) released into the water column, and used those data to parameterise a biophysical dispersal model. Over 0–0.4 m in tanks, larger stages sank more quickly than smaller stages (1.5 cf. 0.6 cm s^−1^), with sinking speeds of adult lice validated in deeper tanks (0–3 m) and a fjord (~15–24 m). Adult males had the greatest behavioural component, sinking more slowly with host cues present and faster when dead. Detached lice were able to intercept new hosts in a tank (23% within 5 min). We also investigated *Caligus elongatus* , but found their strong swimming not amenable to study. A hydrodynamic dispersal model indicated that detached lice can reach neighbouring cages but rarely neighbouring farms before sinking below cage depth. Simulations comparing farm sites highlighted the influence of site‐specific current conditions on dispersal kernels, and indicated that crowding/handling fish during favourable tides can reduce downstream risk.

## 1 Introduction

Ectoparasitic salmon lice ( *Lepeophtheirus salmonis* ) present a major challenge for sustainable Atlantic salmon ( *Salmo salar* ) farming throughout the north Atlantic and Pacific regions. Salmon lice occur naturally in variable but typically low densities on wild salmonids. However, intensive farming in open sea‐cages allows severe outbreaks that impact the health and welfare of farmed fish, threaten wild salmonid populations by exporting large numbers of infective larvae, and lead to direct and indirect economic losses for farmers (Barrett et al. 2022; Costello 2009; Krkošek et al. 2013; Overton et al. 2019; Thorstad et al. 2015; Torrissen et al. 2013).

Salmon lice attach themselves to the skin or fins of the host and feed on the skin, blood and mucus, in severe cases leading to osmotic stress and other concerns (Grimnes and Jakobsen 1996). Gravid female lice produce fertilised eggs that develop in eggstrings until they hatch into planktonic nauplius larvae. Nauplii disperse and moult into copepodid larvae that infect new hosts, before progressing through five parasitic stages (Hamre et al. 2013). The final three stages—pre‐adult I, pre‐adult II and adult—are mobile, meaning they are free to move around on the surface of the host, and in some cases, transfer from one host to another, especially in aquaculture settings (Birkeland and Jakobsen 1997; Bui et al. 2020; Ritchie 1997; Todd et al. 2005, 2000). Host‐switching may occur voluntarily, such as for mate finding (Bui et al. 2020; Todd et al. 2005), but can also occur following accidental dislodgement, especially during aquaculture procedures involving fish handling that result in fish rubbing against each other, nets, or other equipment, for instance during crowding, size grading, transferring, or harvesting (Berntsen et al. 2018; Bui et al. 2020; Geitung et al. 2025; Thorvaldsen et al. 2019). Moreover, most delousing treatments lead to some lice being detached but not killed, posing a reinfestation risk if not collected. Detached lice are perhaps most likely to attach to a new host within the same sea cage, but more concerningly from an environmental and operational perspective, some may exit the cage and encounter new hosts in neighbouring cages, neighbouring farms, or wild salmonid populations (Birkeland and Jakobsen 1997; Guttu et al. 2025; Ritchie 1997).

The reinfection risk will depend on the number of lice lost to the environment, but also the likelihood of those lice remaining viable and at a suitable depth range long enough to intercept a new host. Recent work shows that large numbers of lice are lost to the environment during crowding and handling (Geitung et al. 2025; Guttu et al. 2025), and that detached pre‐adult and adult salmon lice do remain viable long enough to encounter and attach to a new host, persisting for days to weeks off the host depending on the temperature (Dalvin et al. 2025). However, we otherwise lack basic information on the behaviour of detached mobile stages, including whether mobile stages exhibit active swimming to maintain depth, and whether this behaviour is affected by environmental conditions. Once basic behaviours are quantified, hydrodynamic dispersal models can be used to investigate potential spread from specific cages and farms. Similar models have been used for years to anticipate the spread of larvae (Johnsen et al. 2016; Samsing et al. 2017; Sandvik et al. 2016).

Here, we investigated the sinking rates of detached mobile salmon lice in a controlled environment, including whether vertical behaviour is influenced by salinity levels or the presence of chemosensory host cues. We hypothesised that lice would (i) exhibit some active swimming to maintain depth, (ii) sink more rapidly in brackish water due to lower water density and/or behavioural avoidance and (iii) that lice would swim more actively in the presence of host cues. To test the capacity of detached lice to intercept a new host in optimal conditions, we also released some individuals into a tank containing a small number of salmon smolts. Finally, we used observed sinking rates to parameterise a biophysical dispersal model and simulated the dispersal of detached mobile lice with consideration of site‐specific hydrodynamic conditions at 13 representative farm sites.

## 2 Methods

### 2.1 Behavioural Trials

#### 2.1.1 Sourcing of Lice

All lice used in this study originated from farmed salmon in Masfjorden in western Norway. Some were removed directly from farmed fish during routine louse counts within sea‐cages and held in incubators (as described in Hamre et al. 2009) at the Matre Aquaculture Research Station until required. The incubators received a constant flow of seawater maintained at 9°C and 34 ppt salinity. These farm‐sourced lice were generally used within 0–2 days of collection (maximum 7 days). The remaining lice were sourced from a captive population maintained on host fish at the research station. The captive population was established by collecting fertilised eggs from farmed fish in Masfjorden in Jan 2023, shortly before the experiment. The eggs were incubated at 9°C and 34 ppt salinity until infective copepodid larvae had developed, which were then used to infect a tank‐based salmon population. The infection was performed by lowering the water level to 20 cm and pouring the larvae into the tank. The water inlet and outlet were maintained, and the water level in the tanks returned to normal within 10 min. Dissolved oxygen did not fall below 80% saturation. Water temperature was adjusted to obtain the required developmental speed (Hamre et al. 2019) and varied from 9°C to 15°C (34 ppt salinity throughout). As the cohort of lice developed, individual fish were netted out of the tank as required, euthanised with a blow to the head and placed in seawater. Mobile lice were gently removed with forceps, sorted by sex and stage in petri dishes (9°C, 34 ppt salinity), and then held in incubators for up to 2 days before use in the behavioural trials.

To supplement the study of salmon lice, a small number of *Caligus elongatus* sea lice were also sourced from a laboratory strain reared on lumpfish ( *Cyclopterus lumpus* ) at the University of Bergen (outlined in Hamre et al. 2024). The strain originated from wild specimens collected in 2019 from Senja, Northern Norway. The host lumpfish were infested as for salmon lice, with the host population maintained at 9°C and full salinity (~34 ppt) until adult lice had developed, at which point the hosts were anaesthetised and adult lice were carefully removed using forceps.

Host salmon and lumpfish were monitored daily to ensure good welfare in line with permission conditions (Norwegian Food Safety Authority application numbers 26,020, 203,748 and 275,263).

#### 2.1.2 Experimental Design and Protocol

We investigated the vertical behaviour of detached mobile stages by releasing individuals into the water column, under controlled conditions, and quantifying their sinking rate. These trials were conducted at 9°C to match the temperature of the incubators, while several other factors were varied to assess effects on sinking rate: salinity (24 or 34 ppt), presence of chemosensory host cues (seawater taken from either an ambient or salmon‐conditioned source), and presence of behaviour (live or dead lice). Dead lice were produced by transferring individuals from the incubator to a 70 mL plastic specimen jar, together with a small volume of water, and then warming the jar to 38°C for 1 min using a water bath (sufficient to kill all individuals while minimising potential effects on buoyancy). Individual lice were treated as experimental replicates, and thus were only used once as live individuals and once (at most) as dead individuals (see Model 1).

The sinking rate of every mobile stage (pre‐adult I to adult) was tested over a 0–40 cm depth range in white high‐density polyethylene buckets (38 L, diameter of 34 cm at half depth, Rubbermaid Brute). Before each trial, the bucket was filled to 40 cm with seawater of the specified salinity, with or without host cues depending on the treatment group, and allowed to stand for 10 min to reduce turbulence. Lice were then removed from an incubator using a disposable pipette with an enlarged opening to prevent injury and injected into the centre of the bucket, just under the surface (0–1 cm depth). The behaviour of the louse was observed, and the time taken to reach the bottom of the bucket was recorded. The sinking rate was calculated by dividing the elapsed time by the depth of the bucket. In rare cases, sustained horizontal swimming caused a louse to reach the wall of the bucket before the bottom. If the louse clung to or repeatedly contacted the wall, the trial was terminated, and the sinking rate was calculated based on the elapsed time and depth at which it first contacted the wall.

Sinking rates of adult lice were also tested over 0–300 cm within a cylindrical concrete tank (6 m diameter) filled to 300 cm depth with 34 ppt seawater at 9°C. The tank was located outside the station, but had a permanent tarpaulin covering that blocked natural light, with constant overhead lighting (white LED work light, 5000 lm, 4000 K, 47 W: biltema.no) underneath the tarpaulin providing 100 to 5 μE m^−2^ s^−1^ (LI193SA light sensor, Licor) from 10 to 300 cm depth. The seawater flow was shut off ~1 h before commencing the trial to reduce turbulence, at which point adult male and female lice were transferred from incubators into specimen jars (containing water from the incubator) and then released one by one into the tank. Individuals were then visually tracked using binoculars (Razor UHD 10 × 42: Vortex Optics), and the time taken to reach the bottom of the tank was recorded. No individuals reached the tank wall, so their sinking rate was calculated based on the time at which they first contacted the tank bottom.

To validate findings from the bucket and tank trials (above) over greater depths (and pressure) in a wild setting, 5 adult female lice (1 with egg strings), sourced from the same origin as above, were also released into an open fjord environment (Masfjorden) in March and April 2024. Each louse was transported from an incubator to the fjord in an individual plastic vial. Due to the presence of a strong brackish layer at the surface, a scuba diver carried each vial to 15.5–18.0 m depth to avoid the pycnocline and turbid conditions. There was an average salinity and temperature of 33 ppt and 6°C in this water mass. Each louse was released from a vial and followed by a diver (~2 m away/above to avoid influencing its behaviour) until it reached the seafloor at 18.0–24.4 m depth, at which point the depth range and elapsed time were noted. Sinking rates in the fjord are compared graphically to those in buckets and tanks, but are not formally analysed due to the small sample size.

We then investigated the capacity of detached mobile lice to intercept and reattach to a free‐swimming host in a tank environment. Salmon lice (*n* = 22) and *C. elongatus* (*n* = 5) were removed from host fish using forceps, transferred to a pipette or small vial, and released individually into a 325 L white fibreglass tank (90 × 90 cm, filled to 40 cm depth) with circular flow of 34 ppt seawater at 9°C, and 2–3 salmon smolts swimming freely. Lice were released just above the water's surface and then visually tracked until either an outcome was observed, > 5 min elapsed, or the individual was lost from sight. Possible outcomes included the louse attaching to a host, attaching to the tank sides or bottom, or exiting via the drain at the bottom of the tank. The findings are summarised graphically but not formally analysed due to the small sample size.

#### 2.1.3 Statistical Analysis

Three linear models were fitted to data from the bucket and tank trials (refer to Table 2 for model structures and significance of predictors). Model 1 was designed to test effects of salinity and life stage (‘Salinity’ and ‘Stage’ factors, respectively) on sinking rates, while also distinguishing between the effects of louse swimming behaviour vs. buoyancy by comparing the sinking rates of live and dead lice (‘Status’ factor). The model included full interactions between Status, Stage and Salinity. An additional factor (‘Source’) was specified, without interactions, to account for potential differences in the sinking rates of lice raised on hosts in flowthrough tanks at Matre vs. those obtained from sea‐cages at the nearby Knappen Solheim farm. The model was fitted using the *lm* function in *R* (R Core Team 2023), the suitability of the model was assessed using residual plots, and the results were interpreted via type II analysis of variance tables using the *car* package (Fox and Weisberg 2019), followed by extraction and plotting of estimated marginal means using the *ggeffects* and *ggplot2* packages (Lüdecke 2018; Wickham 2016). Where required, pairwise post hoc comparisons were performed with consideration of interaction effects using the *emmeans* package (Lenth 2020). Model 2 was primarily designed to test whether the presence of salmon host cues altered the vertical swimming behaviour of detached mobile salmon lice. The model was specified with factors for the presence or absence of host cues (‘Conditioning’ factor), Stage and Source, with a Conditioning × Stage interaction term to test whether certain stages were more responsive to host cues than others. Model 3 was designed to validate sinking behaviour over 0–40 cm in the bucket by comparing stage‐specific sinking rates in the bucket to sinking rates observed over 0–300 cm in the concrete tank. Conditions not tested in the concrete tank were removed from the dataset (pre‐adult stages, brackish water, dead lice), and the model was specified with factors indicating the use of a bucket or tank (‘Environment’), Stage, and a Environment × Stage interaction. Models 2 and 3 were each fitted and evaluated as per Model 1.

### 2.2 Dispersal Modelling

#### 2.2.1 Modelling Approach

Dispersal of detached mobile salmon lice was modelled using Lagrangian Advection and Diffusion Model (LADiM) particle tracking software (https://github.com/bjornaa/ladim). The three‐dimensional current fields needed for the particle tracking are provided by the ocean model system NorFjords‐160, which is based on the hydrodynamic ocean circulation model Regional Ocean Modelling System (www.myroms.org: Haidvogel et al. 2008; Shchepetkin and McWilliams 2005). NorFjords‐160 is a modification of the NorKyst‐800 system (Albretsen et al. 2011) with a horizontal resolution of 160 m × 160 m and hydrodynamic forcing on the boundaries from NorKyst‐800. The model is run with 35 depth levels in a s‐coordinate system, with hourly outputs to the particle tracking model. For details, see (Asplin et al. 2020).

Particles (lice) were programmed to behave according to an individual‐based model, where the particles sink at a constant velocity corresponding to the mean sinking rate of a given life stage observed in the present study (live lice in full salinity water without host cues added). Dispersal was modelled for the stages with the highest and lowest sinking velocities (pre‐adult I: 0.6 cm s^−1^; adult female: 1.5 cm s^−1^: Table 1). Due to sinking rates (maximum 2 days to reach the ocean floor), survival time was not a limiting factor. The lice were advected by the Euler‐Forward advection scheme with a horizontal diffusion of 0.1 m^2^ s^−1^ and a timestep of 60 s. The dispersion results were outputted hourly and aggregated onto a grid of 32 m × 32 m. Lice positions are shown as the vertical grid cell where the particles reach 30 (60) m depth, thus showing the horizontal extent of the dispersal of lice at 30 (60) m depth. The number of lice presented is a relative number as it depends on the amount of particles (lice) released. Thus, the dispersal results show the potential for spreading of the detached salmon lice, not absolute numbers.

**TABLE 1:** Summary of observed sinking rates of salmon louse life stages according to the test environment (Env.), live/dead status, salinity and chemosensory cues (conditioning).

Env.	Stage	Status	Salinity (ppt)	Conditioning	*n*	Mean sinking rate (cm/s)	SD	SE
Bucket	Pre‐adult I	Alive	24	Ambient	23	0.79	0.17	0.03
			34	Ambient	39	0.57	0.18	0.03
				Salmon	28	0.49	0.22	0.04
		Dead	24	Ambient	20	0.51	0.25	0.06
			34	Ambient	50	0.50	0.21	0.03
	Pre‐adult II female	Alive	24	Ambient	21	0.85	0.30	0.06
			34	Ambient	60	0.65	0.25	0.03
				Salmon	42	0.66	0.26	0.04
		Dead	24	Ambient	21	0.80	0.21	0.05
			34	Ambient	58	0.61	0.27	0.04
	Pre‐adult II male	Alive	24	Ambient	27	0.67	0.19	0.04
			34	Ambient	53	0.57	0.22	0.03
				Salmon	12	0.53	0.23	0.07
		Dead	24	Ambient	25	0.44	0.17	0.03
			34	Ambient	61	0.51	0.28	0.04
	Adult male	Alive	24	Ambient	22	0.89	0.36	0.08
			34	Ambient	68	0.76	0.47	0.06
				Salmon	39	0.52	0.39	0.06
		Dead	24	Ambient	22	1.11	0.34	0.07
			34	Ambient	84	1.05	0.16	0.02
	Adult female	Alive	24	Ambient	27	1.65	0.42	0.08
			34	Ambient	62	1.55	0.61	0.08
				Salmon	32	1.50	0.31	0.05
		Dead	24	Ambient	19	1.73	0.39	0.09
			34	Ambient	61	1.54	0.37	0.05
	Adult female (ovi.)	Alive	24	Ambient	4	1.38	0.19	0.09
			34	Ambient	10	1.79	0.55	0.18
				Salmon	11	1.69	0.38	0.11
		Dead	24	Ambient	4	1.23	0.05	0.03
			34	Ambient	10	1.54	0.28	0.09
Tank	Adult male	Alive	34	Ambient	5	0.58	0.35	0.16
	Adult female	Alive	34	Ambient	7	1.36	0.48	0.18
	Adult female (ovi.)	Alive	34	Ambient	12	1.63	0.19	0.06
Fjord	Adult female	Alive	33	Ambient	4	1.25	0.34	0.17
	Adult female (ovi.)	Alive	33	Ambient	1	1.37	—	—

#### 2.2.2 Dispersal Simulations

Dispersal of detached mobile lice was simulated at 13 farm sites. Ten particles were released from random locations within each cage of the farm every 30 min at depths of 0–2 m, from January through December 2022 (the most recent year with complete modelled current fields). Seven of the 13 farms (Otervika, Klungset, Hestabyneset, Gjengane, Farmannsøya, Fugløya, Ringholmen) were chosen as they used submerged farming methods during 2022–2024, with a net bottom as deep as ~60 m and a net roof with air‐dome for swim bladder refilling (Oppedal et al. 2020; Warren‐Myers et al. 2024, 2022). This new cage design (Nautilus: https://www.akvagroup.com/nautilus) represents some of the deepest cages used in the industry at present and thus may have greater potential to encounter detached mobile salmon lice as they drift and sink from other cages. The same 7 sites are also known to have high infestation pressure (Lakseluskartet 2024), high lice levels and relatively frequent delousing treatments (BarentsWatch 2024). The remaining 6 sites represent the upper and lower extremes of current speed among Norwegian salmon farms, including the 3 farms (Kariskjæret, Brattholmen, Torgerhaugen) with the highest mean current speeds during 2022, and 3 farms (Båfjorden, Kilaneset, Ommundsteigen) with the lowest.

#### 2.2.3 Tidal Experiments

To investigate the variation in dispersal of lice during different times in a tidal cycle, two model setups for the Otervika farm were developed. The strongest tidal currents in a fjord or fjord mouth typically occur between high and low tide; the maximum incoming tidal current occurs when the tide is rising, while the maximum outgoing tidal current occurs when the tide is falling. The Otervika farm is aligned with the main current direction and has incoming and outgoing currents associated with the tides. We extracted the tidal components (u_tide_) of the along‐fjord surface current (u) at the farm location using a Python implementation (https://github.com/wesleybowman/UTide/) of the Matlab package UTide (Codiga 2011). A high and low tidal experiment was designed by finding all times with maximum incoming and outgoing tidal currents where the residual current (u—u_tide_) is below a threshold of 0.04 ms^−1^ during the year 2022. The threshold was applied to capture conditions when the tidal current is more important than the wind, pressure, or stratification‐driven current, and resulted in 225 instances with incoming current conditions where the tidal current dominated, and 261 instances with outgoing current conditions. For each instance, 100 particles were released from each cage at Otervika at 0–2 m depth. Reported results are for particles released from the northernmost cage. A sinking velocity of 0.6 cm s^−1^ was used, corresponding to the mean sinking rate of live pre‐adult I lice in full salinity conditions without the addition of host cues (Table 1).

## 3 Results

### 3.1 Behavioural Experiments

#### 3.1.1 Sinking Rates

**FIGURE 1:**
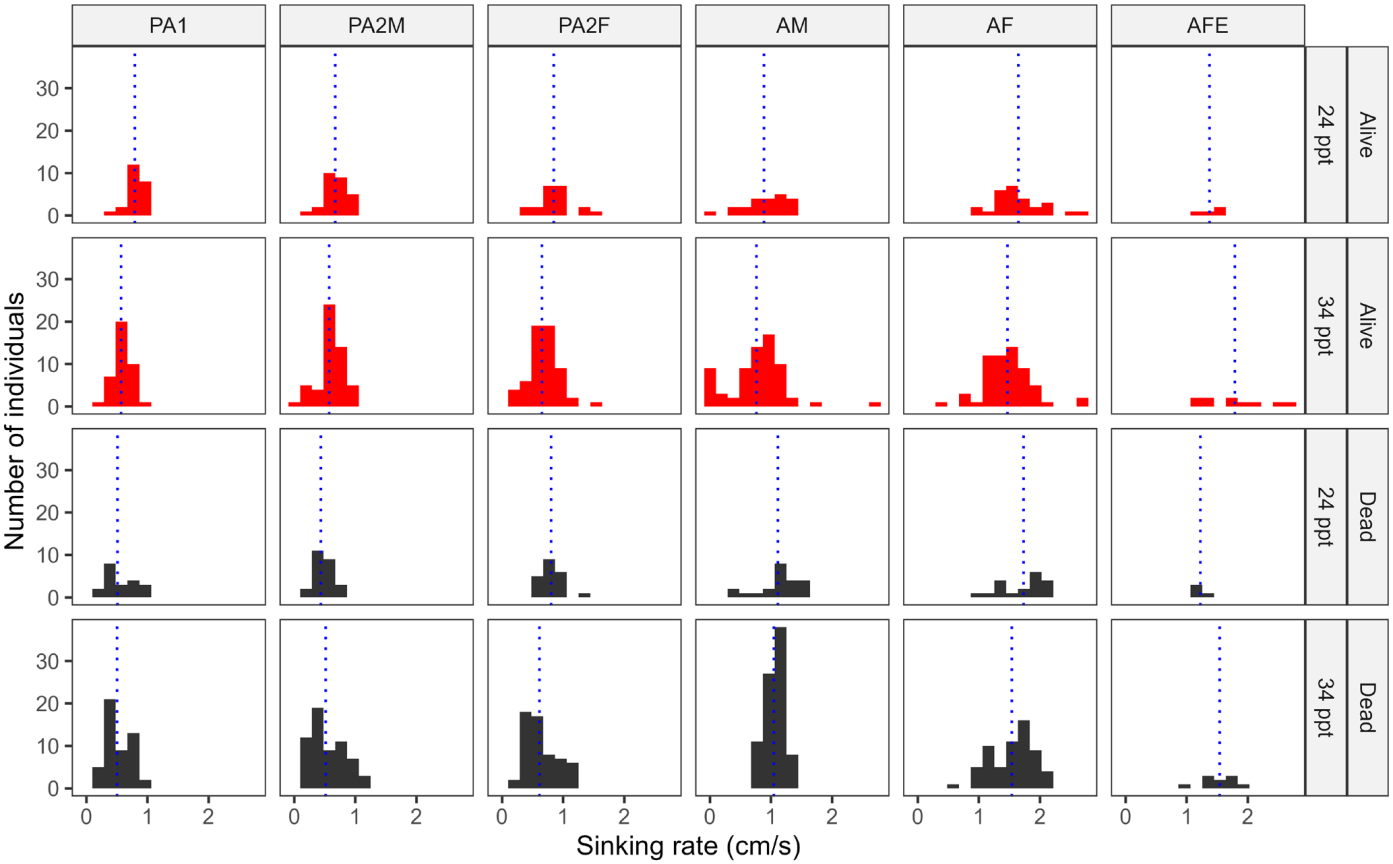
Histograms comparing the distribution of sinking rates of live and dead mobile salmon lice (PA1 = pre‐adult I, PA2M = pre‐adult II male, PA2F = pre‐adult II female, AM = adult male, AF = adult female, AFE = gravid adult female) in brackish or full salinity seawater (24 or 34 ppt). Sinking rates were measured from 0 to 40 cm depth in a white bucket with artificial white lights overhead. The vertical dashed line shows the mean sinking rate corresponding to each panel.

**FIGURE 2:**
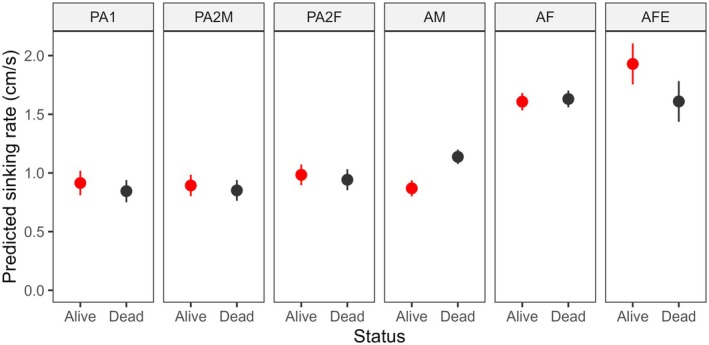
Predicted sinking rates (estimated marginal means ±95% confidence intervals from Model 1) of live and dead mobile salmon lice (PA1 = pre‐adult I, PA2M = pre‐adult II male, PA2F = pre‐adult II female, AM = adult male, AF = adult female, AFE = gravid adult female). Sinking rates were measured over 0–40 cm depth, with predictions conditional on 34 ppt seawater without the addition of any host cues.

**FIGURE 3:**
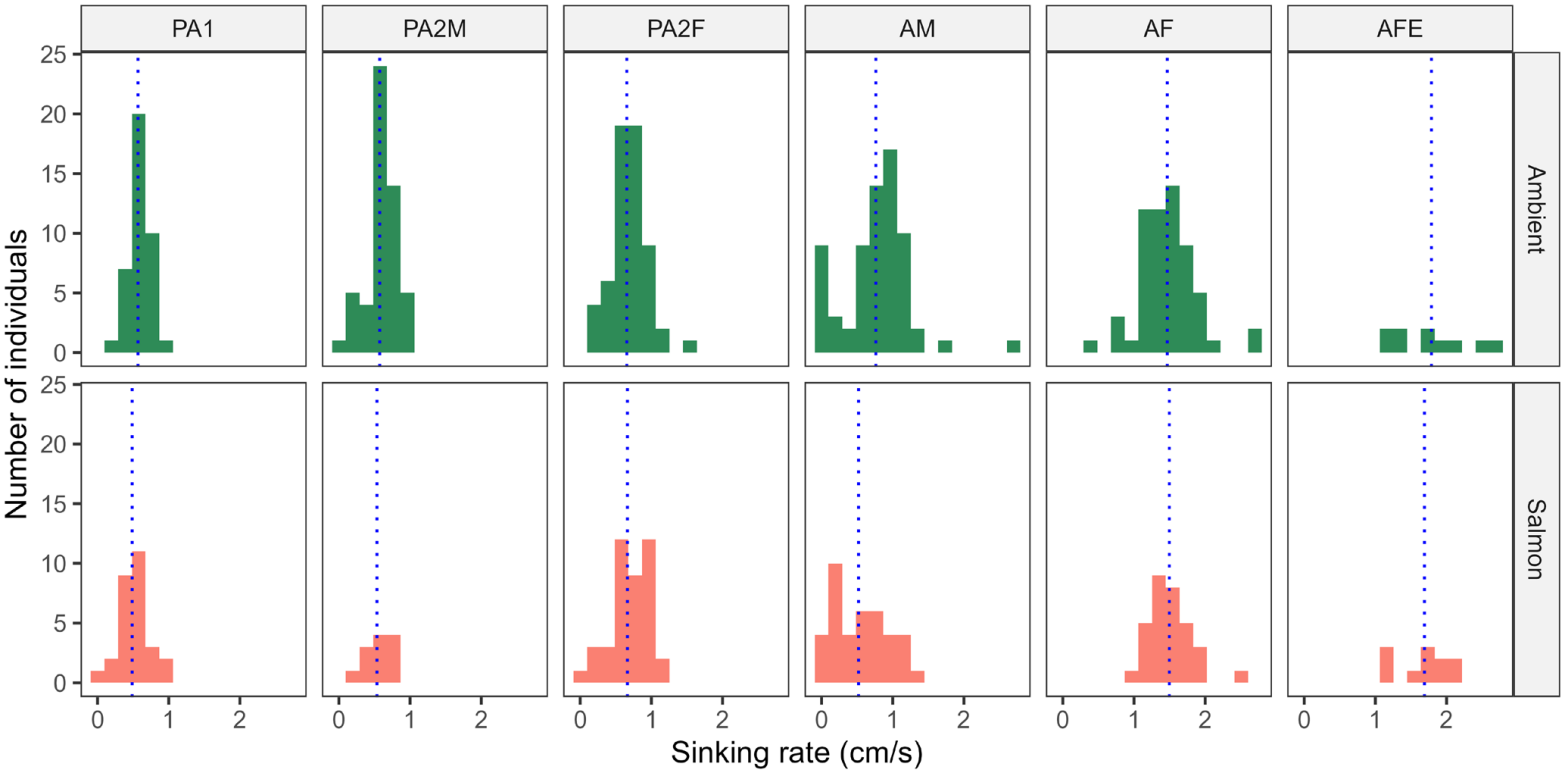
Histograms comparing the distribution of sinking rates of live mobile salmon lice (PA1 = pre‐adult I, PA2M = pre‐adult II male, PA2F = pre‐adult II female, AM = adult male, AF = adult female, AFE = gravid adult female) with or without the addition of salmon‐conditioned seawater to provide a chemosensory host cue. Sinking rates were measured from 0 to 40 cm depth in a white bucket with 34 ppt salinity and artificial white lights overhead. The vertical dashed line shows the mean sinking rate corresponding to each panel.

**FIGURE 4:**
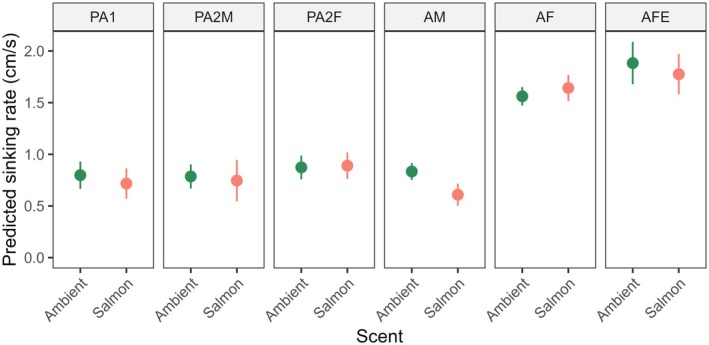
Predicted sinking rates (estimated marginal means ±95% confidence intervals from Model 2) of live mobile salmon lice (PA1 = pre‐adult I, PA2M = pre‐adult II male, PA2F = pre‐adult II female, AM = adult male, AF = adult female, AFE = gravid adult female) with or without the addition of salmon‐conditioned seawater to provide a chemosensory host cue. Sinking rates were measured from 0 to 40 cm depth in a white bucket with 34 ppt salinity and artificial white lights overhead.

**FIGURE 5:**
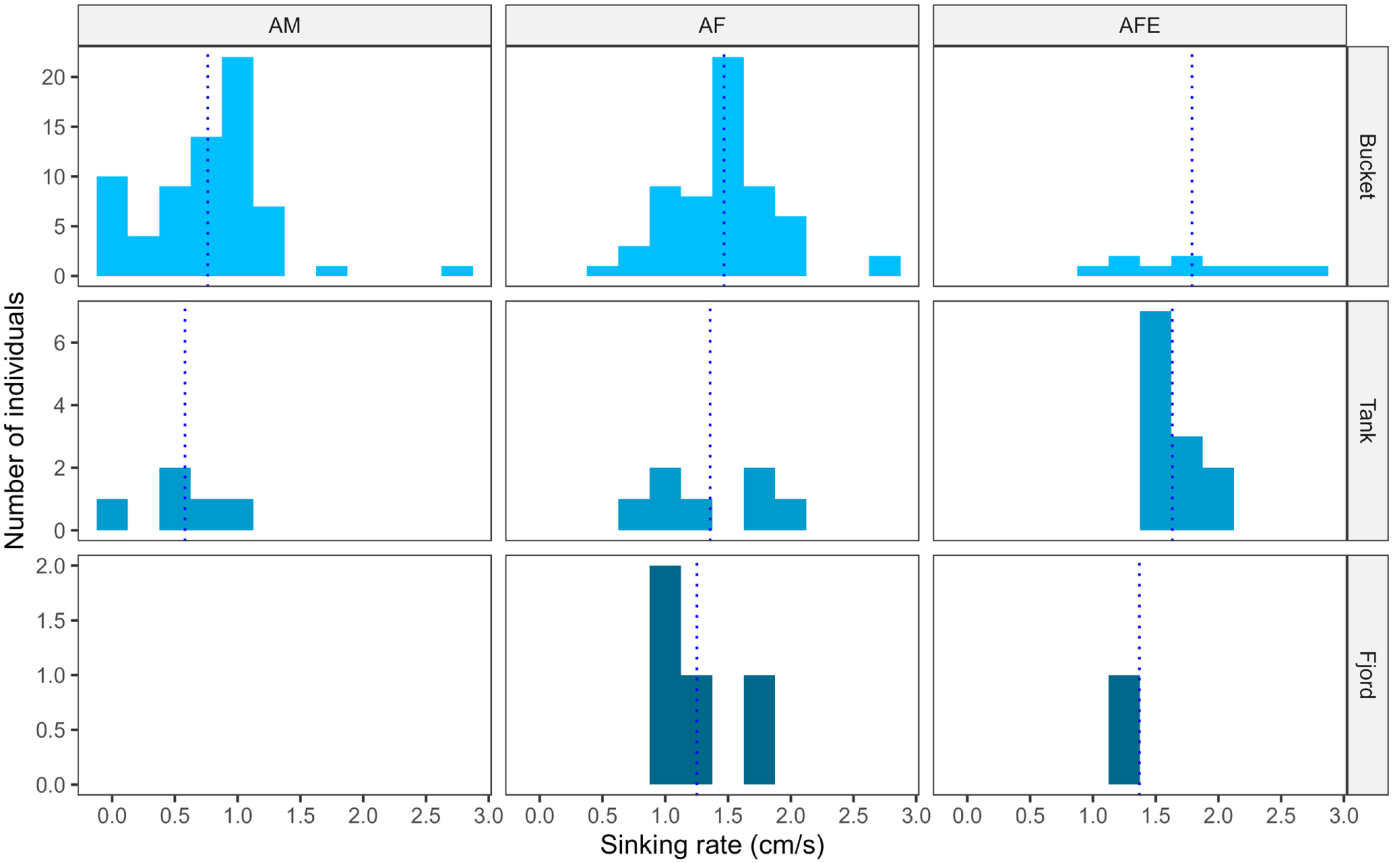
Histograms showing similar sinking rates among live mobile salmon lice released into 40‐cm deep buckets, a 3‐m deep concrete tank, or > 15 m depth in a fjord. Only adult stages were released into the concrete tank: AM = adult male, AF = adult female, AFE = gravid adult female. Bucket and tank trials were conducted with 34 ppt salinity and 9°C, without the addition of any host cues, and with artificial overhead lighting (white light). The fjord trial was conducted at 33 ppt and 6°C. The vertical dashed line shows the mean sinking rate corresponding to each panel.

All mobile life stages of the salmon louse were negatively buoyant in both brackish and full salinity seawater, whether alive or dead (Figure 1). However, sinking rates varied significantly between life stages (Model 1: Tables 1 and 2). In general, the larger stages sank more quickly, with gravid adult females being the fastest, followed by non‐gravid adult females (Figure 2). Most stages exhibited weak, if any, vertical swimming behaviour. Anecdotally, pre‐adult stages generally sank passively or swam erratically and intermittently, while adult males tended to swim upwards or diagonally upwards to maintain depth, and adult females either sank passively or, less often, swam downwards. During trials with live lice in the bucket, adult males were also the most likely to reach the bucket wall, which required the individual to swim > 16 cm horizontally before reaching 40 cm depth (slope < 2.5:1, achieved by 8.1% of adult males, 4.4% of pre‐adult II males, and < 2.5% of other stages). These differing behaviours presumably contributed to stage‐specific differences in sinking rates between live and dead lice (Model 1: Tables 1 and 2), such that live and dead pre‐adults sank at similar rates on average (*p* > 0.05), while live adult males sank slower than dead adult males (*p* < 0.0001), and gravid adult females sank faster when alive than dead (*p* = 0.01) (Figure 2). There was a significant interaction effect between salinity and stage (Model 1: Tables 1 and 2); overall, lice sank faster in brackish water whether alive or dead, although the effect was relatively weak and inconsistent among stages (Figure 1). The addition of salmon‐conditioned seawater to provide chemosensory host cues increased the vertical swimming behaviour of adult males, causing them to sink more slowly in the presence of host cues (*p* = 0.0007) (Figure 3), while all other stages showed little or no change in behaviour (*p* > 0.05 in each case) (Model 2: Table 2, Figure 4). Sinking rates of adult stages over 0–40 cm in a bucket were similar to those observed over 0–300 cm in concrete tanks (Figure 5), with no significant effect of the test environment detected according to Model 3 (Table 2). The sinking rates of adult lice tracked in the fjord also fell within the same range (Figure 5), suggesting that the patterns observed in the bucket trials may be applied, with some caution, over a greater depth range in the field.

**TABLE 2:** Analysis of variance tables (type II) corresponding to Models 1–3.

Term	SS	df	*F*	*p*
*Model 1*				
Status	0.01	1	0.10	0.72
Stage	74	5	189	< 0.0001
Salinity	3.9	1	50	< 0.0001
Source	12	1	151	< 0.0001
Status × Stage	4.8	5	12	< 0.0001
Status × Salinity	0.2	1	3.0	0.083
Stage × Salinity	2.1	5	5.4	0.0001
Status × Stage × Salinity	0.5	5	1.2	0.29
Residuals	64	824		
*Model 2*				
Conditioning	0.3	1	3.1	0.08
Stage	58	5	109	< 0.0001
Source	3.2	1	30	< 0.0001
Conditioning × Stage	1.2	5	2.3	0.041
Residuals	47	441		
*Model 3*				
Environment	0.35	1	1.8	0.17
Stage^a^	24	2	63	< 0.0001
Environment × Stage^a^	0.01	2	0.04	0.96
Residuals	30	156		

*Note:* Model terms include: Status = alive or dead (2 levels); Stage = pre‐adult I, pre‐adult II male, pre‐adult II female, adult male, non‐gravid adult female, gravid adult female (6 levels); Salinity = 24 or 34 ppt (2 levels); Source = sea‐cage or tank‐based host population (2 levels); Conditioning = ambient or salmon‐conditioned seawater (2 levels); Environment = 40‐cm deep bucket or 3‐m deep concrete tank (2 levels).

^a^ Adult stages only.

We were not able to gather usable data on sinking rates of *Caligus elongatus* , as every individual tested (*n* = 10) swam strongly and remained at the surface of the bucket for at least 1 h, at which point we terminated the experiment. All individuals reached the bucket wall at some point, but did not appear to attach to the wall, preferring to continue swimming. Anecdotally, individuals held in incubators tended to exhibit the same surface‐attracted swimming behaviour for hours or days at a time, and were rarely observed resting on the bottom of the incubators. This differed markedly from the behaviour of salmon lice in the same settings, which, unless disturbed, appeared to spend most time resting by attaching to the walls or bottom of the incubator well.

#### 3.1.2 Host Interception and Attachment

**FIGURE 6:**
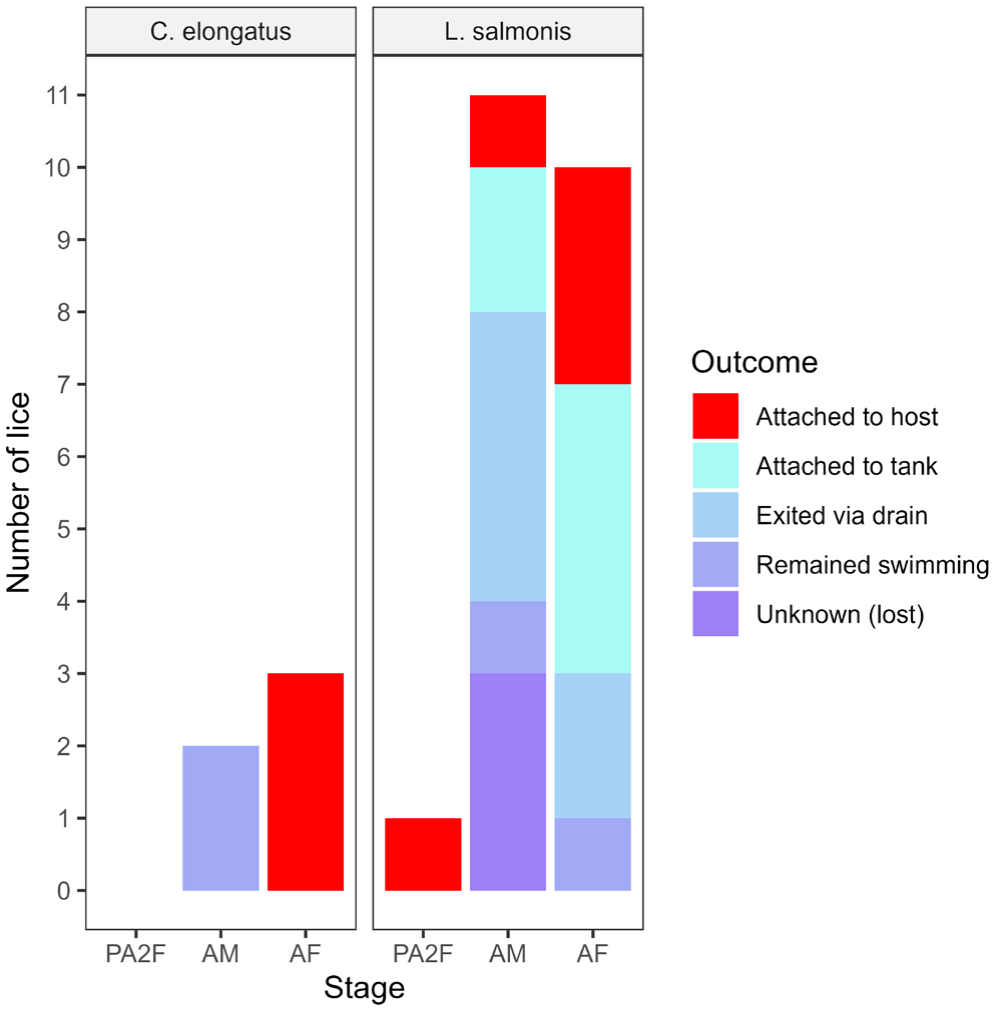
Outcomes from host interception trials in which detached mobile stages of *Caligus elongatus* and *Lepeophtheirus salmonis* were released into a 600‐L tank containing Atlantic salmon smolts. Outcomes are given by species and stage (PA2F = pre‐adult II female, AM = adult male, AF = adult female).

The host interception experiment demonstrated that some detached salmon lice and *C. elongatus* are capable of intercepting and attaching to a new host when given sufficient opportunity. Of the 22 salmon lice released into a 325 L tank containing 2–3 fish, 5 successfully attached to a host within 5 min (23%), while a further 8 attached to another surface or continued swimming actively beyond 5 min and may have eventually intercepted a host if allowed more time (Figure 6). At least 1 individual of each life stage tested successfully attached to a host (1 pre‐adult II female, 1 adult male, 3 adult females). The remaining 9 individuals either exited via the drain (6) or were lost (3, unknown outcome). All 3 adult female *C. elongatus* successfully attached to a host within 5 min, while the 2 adult male *C. elongatus* continued swimming actively just below the surface for at least 20 min (echoing their behaviour in the sinking rate experiment), at which point observations ceased (Figure 6).

### 3.2 Modelled Dispersal

**FIGURE 7:**
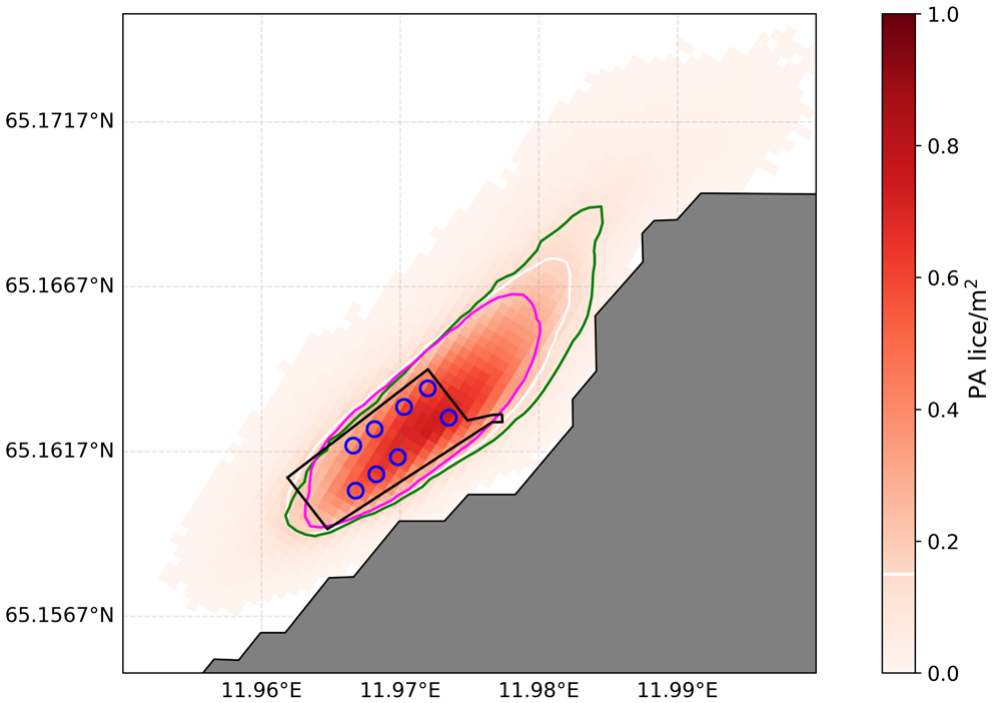
Simulated dispersal of salmon lice with a sinking rate of 0.6 cm s^−1^, representing pre‐adult 1 and pre‐adult 2 male (‘PA’) lice released in batches of 10 lice every 30 min over the year 2022. The red gradient indicates the aggregated density of lice at 30 m depth over the year 2022. The farm area is shown in black, while the blue circles show the position of the cages. The white line denotes 0.15 pre‐adult lice m^−2^ at 30 m depth, the green line denotes 0.15 pre‐adult lice m^−2^ at 60 m depth, and the pink line denotes 0.15 adult female lice m^−2^ at 30 m depth (the latter assuming a sinking rate of 1.5 cm s^−1^).

Overall, particles representing detached mobile salmon lice mainly stay in the vicinity of the farm from which they are released (Figure 7). However, a small number (> 0.15 lice m^−2^) spread up to 1784 m horizontally from the release point before reaching 30 m depth. Because pre‐adults sink more slowly, they dispersed farther than adult females before reaching the depth limit, with a range of 215–1784 m for the maximum spreading distance for pre‐adults and 180–1748 m for adult females (Figure 7, Table 3).

**TABLE 3:** Overview of findings from dispersal modelling runs where 10 particles (lice) are released from 13 farm sites every 30 min through the year 2022.

Farm	Group	Farm layout	Farm direction	Main current direction	Mean (max) current @ 15 m (cm s^−1^)	N. lice release cage (lice m^−2^)	N. lice neighbouring cage (lice m^−2^)	N. lice farm (lice m^−2^)	Distance (m)
Otervika	Sub. farming	2 × 4	NEE/SWW	NEE/SW	7.2 (52.9)	*1.6 (0.8)* **3.5 (2.3)**	*1.6 (0.8)* **3.6 (2.5)**	*0.7 (0.5)* **1.0 (0.9)**	*1128 (1241)* **884 (952)**
Klungset	Sub. farming	2 × 5	NEE/SWW	NEE/SW	8.6 (68.2)	*1.8 (0.9)* **4.1 (3.2)**	*1.6 (0.8)* **3**.**8 (3.0)**	*1.0 (0.6)* **1.5 (1.4)**	*972 (1112)* **828 (890)**
Hestabyneset	Sub. farming	1 × 6	NNW/SSE	NW	9.3 (50.3)	*1.2 (0.6)* **2.5 (1.6)**	*1.4 (0.6)* **2.6 (1.6)**	*0.7 (0.4)* **0.9 (0.7)**	*1268 (1601)* **1006 (1167)**
Gjengane	Sub. farming	1 × 6	N/S	N/S	8.7 (47.0)	*1.0 (0.5)* **2.4 (1.6)**	*1.2 (0.5)* **2.6 (1.7)**	*0.6 (0.4)* **0.9 (0.8)**	*1466 (1537)* **1165 (1236)**
Farmannsøya	Sub. farming	2 × 5	NNE/SSW	NEE/SW	10.5 (58.2)	*0.4 (0.2)* **1.3 (1.0)**	*0.4 (0.2)* **1.0 (0.8)**	*0.4 (0.2)* **0.7 (0.6)**	*1140 (585)* **1039 (1001)**
Fugløya	Sub. farming	2 × 6	NE/SW	WNW/ESE	7.7 (81.6)	*5.8 (1.6)* **12.3 (5.0)**	*2.7 (1.6)* **5.3 (3.9)**	*0.6 (0.2)* **0**.**9 (0.6)**	*1171 (719)* **736 (636)**
Ringholmen	Sub. farming	2 × 8	NE/SW	NW/ESE	12.3 (57.1)	*0.4 (0.2)* **0.9 (0.7)**	*0.4 (0.2)* **1.0 (0. 7)**	*0.1 (0.1)* **0**.**2 (0.2)**	*953 (775)* **921 (1163)**
Båfjorden	Low current	1 × 3	NNE/SSW	N/S	1.1 (8.3)	*7.9* **13.8**	*1.8* **0.7**	*1.1* **1.5**	*215* **196**
Kilaneset	Low current	2 × 2	NNW/SSE	NNW/SSE	1.3 (12.0)	*14.5* **20.2**	*0.8* **1.9**	*3.3* **3.3**	*223* **180**
Ommundsteigen	Low current	2 × 3	NW/SE	WNW/ESE	1.3 (14.0)	*11.3 (7.7)* **20.0 (17.5)**	*7.3 (4.4)* **7.8 (7.7)**	*2.9 (2.6)* **3.2 (3.2)**	*348 (412)* **223 (223)**
Kariskjæret	High current	2 × 5	NE/SW	NEE/SW	20.4 (93.9)	*0.2* **0.7**	*0.16* **0.65**	*0.17* **0.63**	*1017* **878**
Brattholmen	High current	2 × 6	NNE/SSW	N/S	21.5 (137.0)	*0.3* **1.3**	*0.37* **1.63**	*0.5* **1.15**	*1050* **1436 (1499)**
Torgerhaugen	High current	1 × 1	NEE/SWW	NE/SW	26.7 (91.7)	*0.3* **0.8**		*0.23* **0.51**	*1784* ***1748 (1836)***

*Note:* Site characteristics include group (the reason for inclusion in the simulation), farm layout (number of rows × cages per row), farm direction (along the cage rows), main current direction and the mean (max) current at 15 m depth. Lice were released from each cage in the farm, with each release investigated separately. Outcomes are the maximum values resulting from a release from a single cage, and are reported by life stage and depth threshold, with values for 30 m not in parenthesis, values for 60 m in parentheses (omitted if the site is shallower than 60 m), values for pre‐adult I and pre‐adult II males in *italics*, and values for adult females in **bold**. Four outcomes are reported: The relative amount (as a density) of lice reaching the depth threshold within the release cage, reaching the depth threshold within a neighbouring cage, reaching the depth threshold within the farm area, and the maximum distance where > 0.15 lice m^−2^ are found at the threshold depth.

For the 7 sites with submerged cages and moderate current speeds, the range was 953–1466 m for pre‐adults and 736–1165 m for adult females before reaching 30 m depth (Table 3). As expected, the maximum dispersal distance is greater when the depth limit is increased to 60 m, indicating that submerged cages may be at greater risk. Note that in areas where the water depth is between 30 and 60 m, the maximum dispersal distance can be greater at 30 m than at 60 m depth (Table 3) as the bathymetry restricts the dispersal distance. Spreading distance would increase (decrease) with smaller (larger) density limits. The greatest dispersal distances occurred at Gjengane and Hestabyneset, farms with a relatively strong current that is aligned with the direction of the farm (Table 3). Even so, only a negligible number of mobile lice (< 0.001 pre‐adults m^−2^) from Gjengane reached the nearest neighbouring farm of Brattavika at 60 m depth. Otherwise, lice did not spread to neighbouring farms. Ringholmen, where the main current is relatively strong and not aligned with the direction of the farm, had the highest proportion of lice exiting the farm area before reaching the depth limit (Table 3).

A comparison of 3 sites with weak currents (mean 1.1–1.3 cm s^−1^) and 3 sites with strong currents (mean 20–37 cm s^−1^) demonstrates that detached mobile lice spread up to 10× farther when the currents are strong (Table 3), with a much larger proportion of lice exiting the farm area before reaching the depth limit. At the farms with weak currents, 7.9–20.2 lice m^−2^ reach 30 m depth while still within the release cage, compared to < 1.3 lice m^−2^ at the farms with strong currents. Lice released at high current sites were also able to spread to neighbouring farms, with up to 0.06 lice m^−2^ at 30 and 60 m within the neighbouring farm, although this was most likely to occur when releases coincided with the highest current speeds, representing a worst‐case scenario. Contrary to expectations of submerged cages being at greater risk for lice spreading from neighbouring cages, fewer lice are found at 60 m depth than at 30 m depth within the farm itself. The risk of reinfection is therefore smaller in submerged cages within the original farm, although we note that in the rare occurrence of lice reaching neighbouring farms, submerged cages are at higher risk.

**FIGURE 8:**
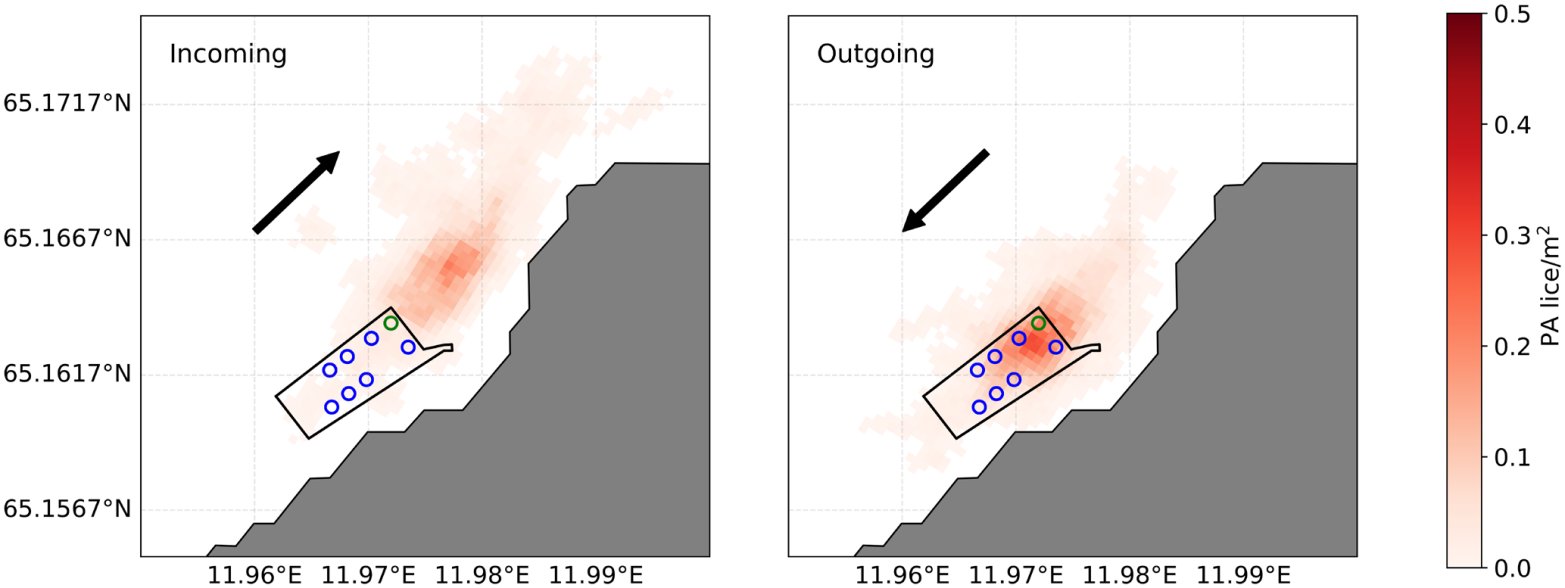
Simulated dispersal of salmon lice according to tidal current conditions. The red gradient indicates the aggregated density of lice at 30 m depth, assuming a sinking rate of 0.6 cm s^−1^ (representing pre‐adult 1 and pre‐adult 2 male lice, ‘PA’) for release times during strong incoming tidal currents (left panel) and strong outgoing tidal currents (right panel). The arrows indicate the general direction of the incoming and outgoing currents. The black line delineates the farm area, while the circles show the position of the cages. The green circle denotes the cage from which the particles were released.

The tidal experiments revealed how dispersal patterns are affected by strong incoming or outgoing tidal currents at Otervika, a farm with cages that are aligned along the fjord (Figure 8). Lice released from the northwestern cages (the fjord end of the farm) during strong incoming tides spread away from the farm, farther into the fjord (Figure 8, left). By contrast, lice released during strong outgoing tides were more likely to reach the depth limit within the farm area (Figure 8, right), potentially infesting fish within other cages at the site. At 30 (60) m depth, the release cage during incoming tidal currents has 76% (63%) fewer pre‐adult lice reaching the depth limit while still within the cage than during outgoing tides. The two adjacent cages at the site have 85 and 88% (82 and 57%) fewer pre‐adult lice during incoming than outgoing tidal currents, while the remaining cages have 86–94% (82–93%) fewer pre‐adult lice during strong incoming tidal currents. Similarly, lice released from the most seaward cage were more likely to remain within the farm area and threaten adjacent cages during incoming tides (results not shown).

## 4 Discussion

Active swimming was observed among all mobile life stages of *L. salmonis* , yet no individuals resisted the effect of gravity indefinitely. The predominant behaviour was passive sinking, sometimes interspersed with short swimming efforts, usually horizontally or toward the surface, and especially among adult males. Sinking rates broadly increased with body size, with adult females significantly less buoyant than other mobile life stages. Some individuals were directly observed intercepting and attaching to new hosts in a tank setting, consistent with previous work that inferred reattachment of detached mobile lice after delousing treatments at commercial farms (Guttu et al. 2025; Olafsen et al. 2021). Moreover, dispersal modelling indicated that pre‐adult and adult life stages typically remain at suitable depths long enough to reach neighbouring cages within the same farm, although only a small proportion of pre‐adult lice have any chance of reaching neighbouring farms before sinking too deep.

### 4.1 Factors Affecting Sinking Rates

Sinking rates varied widely, both within and between life stages. Pre‐adults (both sexes) and adult females were relatively passive, with only small differences in mean sinking rates between live and dead individuals, indicating that morphological or physiological differences (affecting buoyancy and/or drag) may explain most of the variation in sinking rates among those life stages. By contrast, the importance of live/dead status and presence/absence of host cues for adult males reflects a greater influence of swimming behaviour on sinking rates. This is consistent with the current understanding of salmon louse reproductive ecology, wherein females mate singly and can experience mate limitation at low infestation densities (Cox et al. 2017; Ritchie et al. 1996; Stormoen et al. 2013), driving adult males to switch hosts to search for unmated or other reproductively active females and/or avoid competition from other males (Connors et al. 2011; Ritchie et al. 1996; Stephenson 2012). Adult males are presumably adapted to finding and attaching to new hosts as part of this process, and so may pose a greater reinfestation risk than other life stages. Tank‐based experiments have found that pre‐adult I lice will also switch hosts in response to high infestation densities on the initial host, and that pre‐adult II males will do so to search for females (Connors et al. 2011), although it is not clear how widespread these behaviours are. Those stages did not exhibit obvious host‐finding behaviours in the present study, with similar sinking rates with and without host cues present.

We expected that lice would sink faster in brackish water, primarily due to the reduced buoyancy of objects in brackish water, but perhaps also a behavioural component in which lice attempt to avoid an osmotically stressful brackish layer (Andrews and Horsberg 2020) by sinking into a more saline water mass below, as has been observed in larval salmon lice (Crosbie et al. 2019). We did find the expected effect of salinity on sinking rates, but the effect was no more apparent for live lice than dead lice, suggesting that there is minimal behavioural avoidance of brackish water. This may reflect a lack of natural selection for such a response, as mobile lice are typically attached to a host and may be better served by remaining attached to that host for as long as possible, rather than detaching in the hope of finding a new host in a more saline water mass.

Salmon lice exhibited some attraction to surfaces, including the walls of the incubator, bucket or tank. Adult males were the stage most likely to reach the bucket wall and attach (sometimes for a few seconds, sometimes indefinitely), followed by pre‐adult II males. If this is a widespread behaviour in a sea‐cage environment, then it is possible that lice will attach to crowding nets, net pens or other underwater structures. This complicates potential dispersal dynamics by allowing lice to pause their dispersal, or if they attach to moving objects such as the hull of a wellboat, potentially increase their dispersal distance or allow upstream dispersal. A similar risk has also been hypothesised for farm‐associated wild fish based on observations of mobile salmon lice attached to saithe (Bruno and Stone 1990; Lyndon and Toovey 2001), which frequently swim between farm sites (Uglem et al. 2009).

### 4.2 Dispersal Dynamics

At the average salmon farm, mobile lice released in the surface layer of a sea‐cage pose a clear risk to the same or other cages at the same farm, and in areas with strong tidal currents and closely spaced farms, neighbouring farms may also be at risk. However, the tidal experiments highlighted the potential benefits of timing crowding or other handling events to coincide with favourable current conditions. Where logistically feasible (e.g., minimal downtime for delousing vessels), planning around tide times could eliminate the spread of mobile lice between farm sites, even at locations with high current speeds. In practice, delousing in very strong water current conditions is impractical and rarely carried out, meaning existing practices may already be mitigating the risk of between‐farm transmission. Low‐current sites have a larger potential for reinfecting cages within the farm itself, but the risk of transmission to neighbouring farm sites is very low.

The year‐long dispersal experiments included a wide range of current scenarios, including louse particles being released during storms, when farms would likely not be delousing. This will have inflated the maximal dispersal distances observed. However, a counterpoint is that no turbulence or resuspension (lifting lice from the seabed back into the water column) is included in the dispersal modelling. Even without resuspension, incorporation of turbulence could increase the variance (and range) of dispersal distances, thus increasing the maximum dispersal distance, although lice sink relatively quickly and the effect is therefore expected to be small. Accordingly, a month‐long sensitivity test with vertical turbulence of 10^−2^ m^2^ s^−1^, a large number for stratified fjords, found an increase in the maximum dispersal distance of only 1%, with most lice still remaining within the original farm. Resuspension would probably not be influential at deep sites, but at shallower sites, resuspension from the bottom, together with strong mid‐depth currents, could disperse the lice further at depths aligned with open farms.

It is also important to note that the reported density values are relative, depending on the number of lice released at each 30‐min interval. As such, these values should be used to inform the potential dispersal kernel of detached lice, not the precise number of lice that are expected to reach a given farm.

### 4.3 Evolutionary Implications

Most salmon lice in Norwegian waters exist on farmed rather than wild salmon, and as a result, selective processes occurring within farms have the potential to drive the evolution of salmon lice in ways that would be maladaptive on wild hosts (Coates et al. 2021; Dempster et al. 2021). Detachment and host‐switching may be one such example. Wild hosts are expected to select for strong attachment, as lice are relatively safe on the host, and the low density of hosts in the environment means that lice are unlikely to find another if they detach. By contrast, the high density of hosts within sea‐cages will likely reduce the fitness cost of detaching and may therefore select for weaker attachment or voluntary detachment, especially if there are costs to being strongly attached. For instance, strong attachment may come at the cost of mobility on the host (adult males are more mobile on the host and more likely to be dislodged during handling: (Bui et al. 2020; Cox et al. 2017; Stephenson 2012)), while voluntary host‐switching behaviour—to find mates or avoid competition—is more likely to pay off when hosts are abundant. Frequent delousing treatments may also be an important selective process, as lice that remain attached to their host through pre‐treatment crowding are likely to be collected/killed by the delousing treatment, whereas those that are dislodged during crowding can evade the treatment and potentially find a new host in a neighbouring cage. If entering a delousing or harvesting vessel is certain to cause mortality, then being dislodged during pre‐treatment crowding would be beneficial so long as there is a non‐zero probability of finding a new host outside the crowding net. More work is needed to understand these dynamics, but if such evolutionary change is undesirable, it should be a priority to collect lice that detach during crowding (Geitung et al. 2025).

### 4.4 Biosecurity Implications

Lice that feed on and move between multiple hosts could act as a vector for pathogens, either by feeding on infected tissues or by carrying the pathogen externally. Salmon lice have been reported to carry a range of pathogens of concern for salmon aquaculture, including viruses (infectious salmon anaemia virus and salmon alphavirus: Nylund et al. 1991, 1993; Petterson et al. 2009), bacteria (*Aeromonas salmonicida, Tenacibaculum maritimum, Pseudomonas fluorescens, Vibrio* spp.: Barker et al. 2009; Nese and Enger 1993) and microsporidian fungi (*Desmozoon* spp.: Freeman and Sommerville 2009). Jakob et al. (2011) found that salmon lice acquired infectious haematopoietic necrosis virus (IHNV) by feeding on infected hosts, remained virus‐positive for 12 h, and passed on the virus when allowed to attach to a naïve host. Later, Novak et al. (2016) reported that salmon lice acquired the bacterium *Aeromonas salmonicida* by feeding on infected hosts and sometimes transferred it to naïve hosts, although only under certain conditions. Based on available evidence, detached mobile lice should be considered a potential (but perhaps unlikely) vector for salmonid diseases and managed accordingly, especially if dispersal to neighbouring farms is a possibility. Transport on vessels or wild fish could increase the likelihood of dispersal between farms (Bruno and Stone 1990; Lyndon and Toovey 2001; Uglem et al. 2009), although more data are required to assess this risk (Uglem et al. 2014).

## 5 Conclusion

Together, these findings indicate that mobile salmon lice detached during crowding or delousing procedures do pose a risk of reinfestation, but mainly within the farm of origin. Any transmission of mobile lice between neighbouring farms would have biosecurity implications, especially if lice function as a vector for salmon pathogens. Methods to collect lice that detach during farm procedures will reduce the number of lice that escape the farm and thus minimise the associated biosecurity concerns. Based on our preliminary observations of (non)sinking rates in buckets and host interception in small tanks, *Caligus elongatus* has much greater potential for dispersal and reinfestation, although a targeted experiment, perhaps undertaken in the field, is required to better understand the risks posed by this species.

## Author Contributions

**Luke T. Barrett:** conceptualization, investigation, writing – original draft, methodology, visualization, formal analysis. **Mari F. Jensen:** investigation, methodology, writing – review and editing, visualization, formal analysis. **Sussie Dalvin:** conceptualization, funding acquisition, writing – review and editing, methodology. **Frode Oppedal:** conceptualization, investigation, writing – review and editing, funding acquisition, methodology, project administration.

## Conflicts of Interest

The authors declare no conflicts of interest.

## Data Availability

The data that support the findings of this study are available from the corresponding author upon reasonable request.
